# MHC Diversity Across Time and Space

**DOI:** 10.1002/ece3.71371

**Published:** 2025-04-28

**Authors:** Maria Cortazar‐Chinarro, Kayla C. King, Mette Lillie

**Affiliations:** ^1^ MEMEG/Department of Biology Faculty of Science, Lund University Lund Sweden; ^2^ Department of Earth Ocean and Atmospheric Sciences University of British Columbia Vancouver British Columbia Canada; ^3^ Department of Ecology and Genetics, Animal Ecology Uppsala University Uppsala Sweden; ^4^ Department of Zoology University of British Columbia Vancouver British Columbia Canada; ^5^ Department of Microbiology & Immunology University of British Columbia Vancouver British Columbia Canada; ^6^ Department of Biology University of Oxford Oxford UK

**Keywords:** climate change, evolutionary processes, genetic diversity, host–parasite interactions, MHC

## Abstract

Most natural populations are genetically diverse. Understanding how diversity is maintained and distributed across time and space can provide insights into the potential for evolution and extinction of populations. Immunogenetic diversity aids individuals and populations in resisting infectious disease, with many studies linking resistance to genes encoding adaptive immune responses, such as the major histocompatibility complex (MHC) genes. The MHC is particularly important for advancing our understanding of local adaptive processes and host–parasite interactions. Here, we review the emerging work and theory exploring the geographic and temporal patterns of MHC diversity in the wild and how they are shaped by selective and demographic processes. We discuss patterns of variation along latitudinal and altitudinal gradients and place this in the context of Latitude Diversity Gradient and Central Marginal Theories. We emphasize how MHC diversity is often lower at the edges of species distributions, particularly in high‐latitude and high‐altitude regions. We also discuss MHC diversity in natural populations facing climate change. As climate change accelerates and emerging parasites spread, reduced immunogenetic diversity could severely threaten wildlife populations, compromising their resilience and long‐term survival. We propose that including immunogenetic diversity into a larger database of environmental and parasite data would allow biologists to test hypotheses regarding host–parasite coevolution and develop effective measures for conservation.

## Graphical abstract

The article discusses the importance of genetic diversity, particularly in the Major Histocompatibility Complex (MHC) loci, in maintaining evolution and protecting against infectious disease. We review the emerging research on geographic and temporal patterns of MHC diversity in natural populations and highlight the consequences of lower MHC diversity in certain situations, such as at the edge of species distributions or in the face of emerging pathogens. We propose further genomic approaches to comprehensively measure changes in MHC diversity and parasitism in the wild.

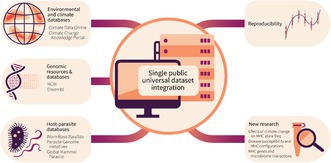

## 1 Introduction

We are facing a severe biodiversity crisis attributable to human activities (Allentoft and O'Brien 2010). Habitat loss and climate change are leading to population declines, loss of genetic diversity and increased extinction risk. Genetic diversity is considered one of the vital pillars of biodiversity and is essential for the capacity of a population to adapt to new environments (Alberto et al. 2013; Frankham 2010; Pauls et al. 2013). The genes of the major histocompatibility complex (MHC) have garnered wide interest for their role in parasite recognition and immune defense at the individual and population level (Sommer 2005). The MHC is typically highly polymorphic, but genetic erosion of the MHC may severely compromise a population's capacity to adapt to changing parasitic environments or the emergence of novel parasites.

In this review, we summarize the key features of the MHC and discuss the dynamics influencing MHC diversity across spatial and temporal scales. We also explore these dynamics in the context of future climate change. Finally, we present how to integrate immunogenetic, environmental, and parasite data across taxa to test hypotheses and uncover the spatial and temporal patterns. Understanding overarching geographical and long‐term temporal patterns has exceptional merit for conservation biology, informing appropriate actions to limit parasite spread within and across populations (Ekroth et al. 2021; Langwig et al. 2015; Smith et al. 2009).

### 1.1 MHC Diversity and the Processes Maintaining Variation

MHC is a multi‐gene family found in all jawed vertebrates, which is considered the most variable set of genes in the genome (Hood et al. 1983; Hughes 2002; Kaufman et al. 1985; Klein 1975; Rock et al. 2016). The MHC plays a crucial role in parasite recognition and activation of the immune response. Each MHC class I and II protein binds to a specific set of peptides, with distinct MHC proteins recognizing and interacting with different antigenic peptides (Kaufman et al. 1985). The high genetic diversity in the MHC region is concentrated in the domains that encode antigen‐binding sites of the classical MHC class I and class II molecules (Klein 1975). MHC class I is predominately expressed on nucleated cells and is involved in the surveillance of intracellular parasites, such as viruses. MHC class II is expressed on specialized immune cells, such as B‐cells and dendritic cells, and is thus involved in the surveillance of extracellular parasites, such as bacteria and macro‐parasites (Klein 1979). The high variation in MHC molecules is directly linked to their ability to bind a wide array of antigens from various pathogens—viruses, bacteria, and macro‐parasites—thereby enhancing recognition by the host immune system (Kaufman et al. 1985). MHC diversity is further amplified by copy number variation within species, such that multiple gene copies can be found across individuals (Minias 2024). This variation expands the repertoire of antigen presentation and parasite recognition of individuals (Bentkowski and Radwan 2019). It also creates a challenge, however, to accurately characterize MHC diversity by for example, amplicon sequencing or whole genome sequencing by short‐read sequencing technologies, as sequences cannot be assigned to specific MHC loci (Babik 2010; Lighten et al. 2014; Peel et al. 2022). Many studies on MHC diversity focus on the specific exon or exons involved in peptide binding and analyze MHC genotypes, supertypes, or heterozygosity in the context of for example, molecular evolution, population genetics, conservation biology, disease associations, and mate choice (reviewed in Cheng et al. 2022). MHC diversity in the context of infection resistance has been studied in amphibians, birds, reptiles, and mammals (Kosch et al. 2022; Nash and Ryan 2023; Savage and Zamudio 2011; Schmid et al. 2023; Vinkler et al. 2022).

Host parasite resistance and fitness are closely related to the variability of MHC molecules (Vinkler et al. 2022). Sedge warblers ( *Acrocephalus schoenobaenus* ) with many MHC class I supertypes have higher resistance to avian malaria (Biedrzycka and Radwan 2008). Associations between specific MHC alleles and avian malaria prevalence have also been reported in other Passeriformes (Bonneaud et al. 2006; Westerdahl et al. 2005). In amphibians, specific MHC class II exon 2 haplotypes in common toads ( *Bufo bufo* ) are associated with individual survival rates to infection by the deadly chytrid fungus, *Batrachochytrium dendrobatidis*. The survival of infected toads was dependent on MHC haplotype configuration (Cortazar‐Chinarro et al. 2022). Across multiple amphibian species, resistance to chytrid fungus has been linked to the MHC class II pocket‐9 conformation, based on experimental infections and field observations of chytrid‐associated population declines (Bataille et al. 2015).

Parasite‐mediated balancing selection is the main evolutionary pressure maintaining genetic variation at the MHC across populations and between species, with the latter being referred to as trans‐species polymorphism (Charlesworth 2006; Hedrick 1998; Höglund 2009; Sommer 2005). Evidence of parasite‐mediated balancing selection has been found across host taxa, including amphibians (Trujillo et al. 2021), birds (He et al. 2021; Minias et al. 2021), fish (Herdegen‐Radwan et al. 2021), and mammals (Ebert and Fields 2020; Gonzalez 2021; Zhang et al. 2018).

There are three non‐mutually exclusive hypotheses explaining the parasite‐mediated maintenance of MHC polymorphism, namely ‘*Heterozygosity Advantage*’, ‘*Rare Allele Advantage*’ (negative frequency‐dependent selection) and ‘*Fluctuating Selection*’ across landscapes or time (Gibson 2022; Spurgin and Richardson 2010). The ‘*Heterozygosity Advantage*’ hypothesis posits that heterozygous individuals can recognize a larger variety of antigens than homozygous individuals, conferring greater resistance to infection in the former (Lo et al. 2021; Savage et al. 2011). Additionally, individuals with more divergent MHC alleles have been hypothesized to bind and recognize a wider array of antigens, further increasing parasite resistance (*Divergent Allele Advantage*) (Lau et al. 2015; Lenz et al. 2009; Wakeland et al. 1990). The ‘*Rare Allele Advantage*’ hypothesis predicts that a rare or novel allele in a population will confer greater disease resistance, as there is strong selection on parasites from common MHC alleles to overcome resistance (also Bolnick and Stutz 2017; Bourgeois et al. 2021; Takahata and Nei 1990). For example, Trinidadian guppies carrying novel MHC alleles and MHC supertypes experience a 37% reduction in infection intensity of the ectoparasite, *Gyrodactylus turnbulli* (Phillips et al. 2018). Rare allele advantage may also favor old, rare alleles, resulting in a cyclical, co‐evolutionary dynamic in which MHC alleles fluctuate in frequency over time, maintaining diversity (Slade 1992). Finally, the ‘*Fluctuating Selection*’ hypothesis states that spatial and temporal variation in parasite prevalence, diversity and infection intensity generates a heterogenous selective environment, leading to different MHC alleles being favored at different times and/or different places. Overall, this heterogeneity will maintain MHC diversity in host meta‐populations (Hedrick 2002; Spurgin and Richardson 2010). Parasite‐mediated selection under ‘*Fluctuating Selection*’ on the MHC is thus directional, but also variable due to changes in biotic or abiotic environment, as well as chance dispersal and extinction events. Determining the relative roles of these modes of selection is a challenge, as they are not mutually exclusive. All three modes may act to shape overall MHC diversity (Spurgin and Richardson 2010). For example, a role for fluctuating selection has been reported in several species (Osborne et al. 2017; Teacher et al. 2009; Trujillo et al. 2021), and occasionally in combination with either divergent allele advantage (Osborne et al. 2017) or negative frequency‐dependent selection (Teacher et al. 2009; Trujillo et al. 2021). Additionally, geographical variation in modes of selection has been observed, for example in moor frogs ( *Rana arvalis* ). In this species, MHC class II variation was found to decrease in northern populations due to directional selection, and variation was maintained at southern latitudes due to divergent and/or balancing selection (Cortazar‐Chinarro et al. 2018).

MHC diversity can also be shaped by other selective and neutral forces (Wakelin and Apanius 1997). Genetic drift resulting from demographic processes (i.e., post‐glacial recolonization, migration) impacts genetic variation genome‐wide, and has been shown to affect MHC diversity in fragmented and small populations, or at the edge of species distributions (Belasen et al. 2022, 2019; Herdegen et al. 2014; Höglund et al. 2022). MHC‐based mate choice (disassortative mating) may also help to maintain MHC diversity in excess of what is expected under parasite‐mediated selection alone (Ejsmond and Radwan 2015). It has been argued that the combined effects of natural and sexual selection on MHC variants explain how diverse communities of hosts and parasites can be maintained over evolutionary time. That is, even in the face of strong frequency‐dependent selection favoring particular MHC genotypes (Milinski 2006). Models combining the effects of natural selection and MHC‐based disassortative mating in small populations have indicated that while parasite‐mediated selection can accelerate loss of MHC diversity, the stabilizing effects of sexual selection can moderate major fluctuations in allele frequencies and protect functional variants against drift (Jan Ejsmond et al. 2014). Appropriately, the interaction between multiple evolutionary forces contributes to shaping and potentially maintaining important genetic variation in at‐risk populations. Critically, understanding how eco‐evolutionary processes (neutral vs. adaptive) shape genetic variation in wildlife populations is a key challenge. Expanding our study systems to encompass spatial and temporal patterns shows potential to help disentangle the evolutionary dynamics underlying these processes.

## 2 Patterns Across Time and Space

### 2.1 Geographical Effects: Latitudinal and Altitudinal Effects

**FIGURE 1:**
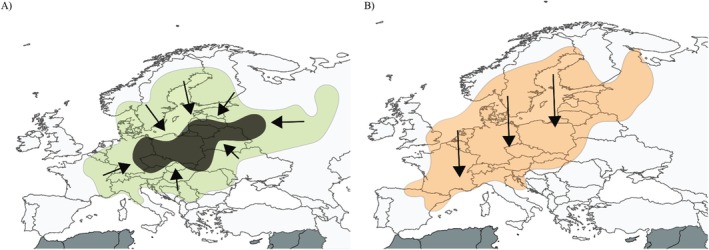
The distribution of genetic variation of a hypothetical species according to the (A) central‐marginal theory (green distribution) and (B) latitudinal diversity gradient trend hypothesis (orange). The black arrows indicate the direction of increased genetic variation.

The genetic diversity of a host species, as well as general species diversity, can vary along a gradient (Cortázar‐Chinarro et al. 2017; Daco et al. 2022; Yiming et al. 2021). Both host immune genetic variation and parasite biodiversity tend to be lower at higher latitudes (Cortázar‐Chinarro et al. 2017; O'Connor et al. 2018; Preisser 2019; Stevens 2006). This pattern has been documented from unicellular to multicellular organisms in concordance with the ‘*Latitudinal Diversity Gradient Trend*’ hypothesis (Miraldo et al. 2016; Preisser 2019; Figure 1). This hypothesis remains relatively untested within individual hosts in relation to parasites (Johnson and Haas 2021). Diversity does, however, tend to increase from the poles to the equator (Adams and Hadly 2013; Pianka 1989). The ‘*Latitudinal Diversity Gradient Trend*’ hypothesis predicts steeper patterns (e.g., clear isolation by distance) in large‐scale gradients (> 1000 km) of latitudinal diversity compared to more localized gradients. Evolutionary mechanisms such as drift, selection, and/or demographic processes (e.g., postglacial colonization events) also contribute to genetic variation distribution around the globe (Cook et al. 2022; Cortázar‐Chinarro et al. 2017; Thörn et al. 2021). Across taxa, genetic variation at MHC molecules can decrease with increasing latitude (e.g., wild ungulates; (Mainguy et al. 2007) mouse; (André et al. 2017) or fish (Dionne et al. 2007)). A similar latitudinal pattern of decreasing MHC genetic variation has been found over the Scandinavian Peninsula in different amphibian species ( *Rana arvalis* ; (Cortázar‐Chinarro et al. 2017), *Bufo bufo* ; (Cortazar‐Chinarro et al. 2019) and *Bufotes viridis* ; (Höglund et al. 2022)). In mountain goats from southwest China, MHC variation has been shown to decrease with increasing altitude (Huang et al. 2015).

Gradients of genetic variation could also be shaped by the *Central‐Marginal Theory* (Eckert et al. 2008) (Figure 1). This theory predicts that genetic diversity is highest at the center of a species distribution, which decreases outwards toward the margins (Eckert et al. 2008; Guo 2012; Thörn et al. 2021). Species range expansion often originates from the core of a species' distribution, where the habitat is optimal for its persistence (e.g., glacial refugia) and progresses toward peripheral regions. Throughout this process, new populations are established through successive population bottlenecks (Singhal et al. 2022). Populations at range margins tend to be smaller and more spatially isolated, becoming subjected to genetic drift and gene flow from the range center. This dynamic often leads to reduced genetic diversity along the expanding range edge, increased population structure, and allele frequency clines centered on the origin of expansion (Peter and Slatkin 2013). For instance, Langin et al. (2017) demonstrated that genetic variation decreased at the range edge of an island scrub‐jay population, with homozygosity increasing toward the island periphery.

### 2.2 Habitat Fragmentation

Long‐term habitat loss and fragmentation, directly caused by human activity, are considered to be significant and widespread drivers of biodiversity loss (Lino et al. 2019). Substantial decreases in population size and species richness as a result may lead to a loss of genetic and immunogenetic diversity (Belasen et al. 2019; Gibbs 2001). The loss of genetic diversity over time due to genetic drift and purifying selection in small populations, together with increased levels of inbreeding, will have serious consequences for disease susceptibility (Zuidema et al. 1996). Habitat fragmentation has resulted in loss of genome‐wide variation as well as MHC diversity in African and Asiatic Cheetahs ( *Acinonyx jubatus* spp) due to genetic drift and inbreeding (Castro‐Prieto et al. 2011; Prost et al. 2022) Similarly, African wild dogs ( *Lycaon pictus* ) in South Africa have lost genomic variation over decades with a parallel decrease of the MHC (Meiring et al. 2022). It has alternatively been shown that immunogenetic variation could be maintained even in populations that experienced loss of genome‐wide diversity by selective processes such as balancing selection (Oliver and Piertney 2012). Functional diversity may therefore be maintained to some extent even in highly fragmented populations (Ciborowski et al. 2017; Escoda and Castresana 2021). Nevertheless, the intrinsic relationship between genome‐wide diversity and adaptive gene variation in endangered species living in severely fragmented habitats remains poorly understood.

Fragmented populations with high inbreeding levels may evolve differently depending on whether they are resistant or susceptible to endemic parasites. “*Resistant fragmented populations*” appear when the contact interaction between hosts infected by the same parasite are limited within a population (Belasen et al. 2019). The reduced contact interaction between hosts will minimize parasite transmission between hosts and an increase of resistant MHC alleles might be generated after directional selection from local parasites (Belasen et al. 2019; Pearson et al. 2009). Conversely, “*Susceptible fragmented populations*” emerge when genetically eroded hosts within a population exhibits a high frequency of MHC alleles that are susceptible to infection, implying strong directional selection on susceptible MHC alleles to local parasites (Belasen et al. 2019; Pearson et al. 2009). Ideally, natural selection would eliminate these susceptible alleles. Other factors, such as co‐infection, may disrupt this process, leading to an increase in susceptible alleles (Cortazar‐Chinarro et al. 2023). The last scenario has been demonstrated in both lab and natural population studies (Ellison et al. 2012, 2011). For example, Ellison et al. (2012) found that both MHC and neutral genetic variation were lost after several generations of selfing in the self‐fertilizing fish *Kryptolebias marmoratus* under experimental conditions. Interestingly, the loss of MHC variation appeared non‐random, suggesting that parasite‐mediated directional selection may favor divergent MHC alleles (Ellison et al. 2012). In general, long‐term fragmentation may negatively affect MHC diversity and geographic distribution, particularly in isolated populations at the edges of species ranges or in habitats severely altered by human activities.

Habitat fragmentation and degradation are expected to also influence parasite prevalence (Perrin et al. 2023). The relationship between anthropogenic disturbances and wildlife disease prevalence is highly variable, with parasite‐mediated selection pressures differing across environments due to variations in parasite ecology, transmission dynamics, and host immune responses (Brearley et al. 2013; Young et al. 2013). Few studies have investigated neutral diversity, MHC diversity, and parasite interactions in disturbed landscapes. In ornate dragon lizards ( *Ctenophorus ornatus* ), individuals showed significant variation in the number of MHC class I alleles. A positive association was found between the average number of MHC class I alleles and tick load in undisturbed reserve populations, but this relationship was not observed in populations from disturbed agricultural areas (Radwan et al. 2014). The observed lower tick load in disturbed landscapes could be the result of weaker population connectivity or a low number of alternative hosts preventing tick transmission. Low transmission would weaken parasite‐mediated selection for MHC variation in these populations (Radwan et al. 2014).

Additionally, variation in parasite‐specific selection pressures was also observed in the Tome's spiny rat ( *Proechimys semispinosus* ), a generalist rodent that inhabits both natural and human‐disturbed landscapes (Fleischer et al. 2024). This study compared neutral genetic diversity, MHC diversity, and parasite prevalence/diversity across landscapes with varying levels of human disturbance, while also considering habitat features that define distinct habitats, as demonstrated in the study by Schwensow et al. (2022). Fleischer et al. (2024) found lower genome‐wide and MHC diversity as well as lower nematode and virus infection rates in forest fragments relative to protected, continuous forests. Associations between MHC alleles and supertypes varied across these landscapes, with most appearing in the continuous forests or protected forest islands (Fleischer et al. 2024). Associations were occasionally in opposite directions, for example, individuals with the MHC class II allele Prse‐DRB*006 were more often infected with *Picobirnavirus* in forest fragments, whereas this same allele was associated with reduced infections by *Picobirnavirus* in protected forests (Fleischer et al. 2024). This study highlights the impact of landscape disturbance on host–parasite interactions and coevolution. Disturbance can lead to altered parasite‐mediated selection and even altered resistance of MHC alleles due to environmental interactions or stressors or other genetic effects.

### 2.3 Temporal Effects

Spatio‐temporal studies generally reveal fluctuations in parasite‐mediated selection and, as a consequence, in associations between MHC diversity and parasites (Piertney and Oliver 2006; Sommer 2005). Long‐term studies are limited. Spatiotemporal studies generally reveal fluctuations in associations between MHC diversity and parasites, as well as the relative evolutionary and selective forces. For instance, the fluctuating selection mechanism was observed in water voles ( *Arvicola terrestris* ); three populations studied over 6 years showed that within‐population, between‐year differentiation for MHC loci was significantly correlated with microsatellites, implying the influence of neutral forces such as migration and drift were shaping temporal changes in MHC diversity (Oliver et al. 2009). Across populations, however, genetic differentiation implied that MHC variation was primarily affected by directional selection and drift, potentially reflecting fluctuating selection (Oliver et al. 2009). Fluctuating selection and rare allele advantage mechanisms were observed in bank voles ( *Myodes glareolus* ). A study of three populations over 11 years revealed that different MHC supertypes were associated with reduced prevalence and abundance of the same helminth across sub‐populations (Migalska et al. 2022). Temporal patterns revealed potential parasite adaptations to common MHC supertypes. Helminth species that have recently colonized or spread in a given subpopulation were more frequently infecting voles with MHC supertypes common in the recent past (Migalska et al. 2022). Additionally, rare‐allele advantage was observed in the feral population of Soay sheep ( *Ovis aries* ) over 20 years of monitoring. The study showed that MHC haplotype D increased overall fitness in males, and this initially rare haplotype underwent directional selection across successive seasons (Huang et al. 2022). Finally, a recent study spanning 21 years of immunogenetic and disease data in a population of meerkats ( *Suricata suricatta* ) revealed strong evidence for rare allele advantage (Mueller‐Klein et al. 2024). In general, MHC allele frequencies fluctuated over time, but specifically, the MHC class II allele Susu‐DRB*13 showed a temporal increase in resistance to tuberculosis with an associated increase in frequency in the population (Mueller‐Klein et al. 2024).

## 3 Abrupt Climatic Changes

Climate change projections estimate that global warming will reach 3.2°C by 2100 under current climate policies, with significant impacts already observed on weather and climate extremes in every region across the globe (Carlson et al. 2022) Warmer temperatures may facilitate the introduction of parasites into new habitats through their vectors (Brooks and Boeger 2019; Epstein 2001; Williams et al. 2002), posing a significant threat to global wildlife health. Indeed, predictions from mammal‐virus models show that range shifts under climate change will significantly increase cross‐species transmission of viruses as species track thermal optima and aggregate at higher elevations (Carlson et al. 2022). In livestock, changes in temperature may alter the geographical ranges of disease vectors, with some expanding (e.g., *Culicoides imicola*) and others contracting (e.g., *tsetse flies*) (Bett et al. 2017). Also, extreme weather events like droughts and El Niño patterns have been associated with outbreaks of diseases such as Rift Valley fever (Bett et al. 2017). In amphibians, rising temperatures have been linked to increased outbreaks of chytrid fungus (*Bd*), contributing to widespread amphibian declines globally (Rollins‐Smith 2017, 2020; Scheele et al. 2019). Heat waves, cold snaps, droughts, heavy rainfall, and tropical cyclones are expected to become more frequent and unpredictable under climate change, which poses a significant challenge for organisms to adapt to these unstable conditions (Smale and Wernberg 2013). Extreme climate events are likely to disrupt physiological, metabolic, respiratory, reproductive, and immunological competence in hosts and intensify the costs of parasitism (Martinez and Merino 2011). While warmer temperatures can strengthen physical barriers to infection, they may also decrease cellular immune responses by modulating both innate and acquired immunity (Scharsack and Franke 2022; Sun et al. 2023).

Host–parasite interactions are impacted by environmental stress, shaping selection on immune genes (Poulin et al. 2024; Wolinska and King 2009). Cold‐adapted species such as the Atlantic cod (*Gadus morhua*), whose immune system functions optimally at 7°C, exhibit severe immunosuppression when exposed to 14°C under experimental infection with the parasite *Loma morhua*, as well as various fungi and bacteria, resulting in 48% mortality (Scharsack and Franke 2022). These findings suggest that cold‐adapted teleosts, such as the Atlantic cod, are highly susceptible to rising temperatures and more vulnerable to infections as temperatures increase (Scharsack and Franke 2022). Likewise, a mass mortality event during the 2003 European heat wave of experimentally reared three‐spined stickleback ( *Gasterosteus aculeatus* ) revealed a strong family effect of parasite load, and those individuals with high parasite load suffered greater mortality (Wegner et al. 2008). In the survivors, researchers were able to detect that within families, an intermediate number of MHC class IIB variants increased survival, but these effects were small compared to between‐family effects, implying the importance of other contributing loci or non‐genetic interactions (Wegner et al. 2008). A study on domestic sheep populations showed that climate warming is expected to intensify infection pressure from gastrointestinal nematodes, driven by shifts in selection pressures within populations (Hayward et al. 2022). High parasite burdens were linked to reduced winter survival, emphasizing the role of strong selection in favoring individuals with more effective immune responses and lower parasite loads (Hayward et al. 2022). These studies highlight the substantial cost of parasitism to hosts during extreme weather and climatic events and the immunogenetic links to survivorship, which result in strong selection of resistance genotypes.

An intensified period of warmer temperatures and extreme climatic events poses a significant challenge for organisms to track the trajectory of evolutionary processes over time. In the central range of a species' distribution and at mid‐latitudes globally, climate change‐driven range expansions may facilitate increased pathogen transmission due to greater taxonomic diversity and higher host contact rates. (Carlson et al. 2022). Genetic diversity in these populations are often high (Guo 2012) and thus standing immunogenetic variation may be available for populations adapt to novel parasites, even in the face of climatic fluctuations. If these species experience population declines and fragmentations due to climate change; however, they are susceptible to loss of immunogenetic diversity. This would increase their risk from increased disease transmission. Populations with limited genetic diversity, such as those at the edges of a species' distribution, may experience heightened susceptibility to parasites under changing environmental conditions, with limited potential for adaptive response. Assuming that parasite distribution is not constant over space at the edge of species distribution, the intensity of selection can vary in space, even at small spatial scales, leading to a more rapid adaptation if parasite pressure becomes significant (King and Lively 2009). Peripheral populations may be disproportionately affected by the anticipated spread of pathogens (Yadav and Upadhyay 2023), particularly vector‐borne pathogens, and subsequent population declines may further erode their genetic diversity. Small populations with limited genetic and immunogenetic diversity may lack the capacity to evolve resistance rapidly enough to match the rapid co‐evolutionary dynamics of pathogens, which have short generation times and large population sizes. Summarily, these complex interactions between various demographic and selective processes present a significant challenge to researchers to predict population susceptibility in the context of climate change and infectious disease. From a conservation perspective, maintenance (or supplementation) of genetic variation, particularly at the MHC and other immune genes, could be particularly important to facilitate rapid adaptation to emerging parasites or altered parasite communities under climate change.

## 4 MHC Diversity in the Wild—Future Directions

To disentangle evolutionary processes (e.g., drift, migration or selection) shaping adaptive genetic variation, the most applied method consists of contrasting spatial patterns of variation between adaptive genes and neutral markers (Babik et al. 2008; Belasen et al. 2019; Cortázar‐Chinarro et al. 2017; Santonastaso et al. 2017; Strand et al. 2012). When selection outweighs demographic processes, the level of differentiation in adapted genes between populations is lower under stabilizing selection, whereas differentiation should be higher under diversifying selection. This pattern suggests adaptation to local parasite faunas (Meyer‐Lucht et al. 2016; Wang et al. 2012). A lack of differentiation between adaptive and neutral markers would instead indicate a dominant role of drift, and demography might be a key contributor to functional diversity at a broad scale (see Cook et al. 2022; Rico et al. 2015, for specific examples). Disentangling the relative contributions of evolutionary forces and demographic events, and understanding how they influence genetic variation in natural populations in the face of infection, remains an unresolved issue. To identify and differentiate between specific selective processes and demographic processes shaping adaptive genetic variation, we propose the integration of specific multidisciplinary data (ecological factors, parasite diversity, changes in allele frequency estimation) to elucidate all potential processes and drivers that contribute to shaping adaptive genetic variation (Spurgin and Richardson 2010) (Box 1).

BOX 1 **Multidisciplinary data (including environmental factors, parasite prevalence data, and allele frequency data) that could help elucidate processes and drivers shaping MHC genetic variation in natural populations.**

(a) *Environmental factors* will give us a better understanding of the habitat characteristics where the organisms live in natural conditions, and will ultimately allow us to explore environmental changes as potential genetic diversity drivers in the nature (Florencio et al. 2014). As an example (Lee and Mitchell‐Olds 2011) estimate the quantitate contribution of environmental adaptation and IBD on genetic variation in *Boechera stricta* , from Arabidopsis genus. They found that the environment had a larger contribution than geography and confirmed the effect of environmental selection (e.g., climate, topography, and latitude) on genetic divergence between East and west lineages. Also, (O'Connor et al. 2020) showed a strong positive selection between precipitation and the number of different MHC‐I alleles. In general, there is a lack of knowledge on how changes in ecological factors over time could shape MHC genetic variation. Therefore, including ecological variables (e.g., temperature, precipitation, topography, canopy cover and water temperature, pH, oxygen saturation when aquatic ecosystems are considered) are needed to better understand changes in functional adaptive genes, like MHC, in future climatic scenarios.

(b) *Parasite diversity, infection intensity and prevalence data*. Novel associations between infection and MHC class II loci have been recently revealed (See for example; (Bataille et al. 2015; Martin et al. 2022; Savage et al. 2019)). Cortazar‐Chinarro et al. (2022) found that the presence of the MHC haplotype Bubu*09 has a negative impact on survival when common toad ( *Bufo bufo* ) individuals were infected with a specific chytrid fungus strain from Sweden. In parallel, haplotype Bubu*02 had a negative impact on survival infected with the UK‐strain, but was marginally beneficial when toads were infected by the Swedish‐strain. Despite the enormous effort on MHC characterization studies, and the association between specific MHC alleles with disease resistance, such associations are rarely investigated (e.g., Martin et al. (2022)). We propose that future studies focusing on MHC functional transcript wide polymorphism, and its comparison to the MHC‐DNA based polymorphism, to better understand how immunogenetics moderates disease in relation to parasite resistance. Finally, screening for infection intensity and parasite prevalence in relation to immune gene variation will be indispensable to investigating disease susceptibility wider patterns and their relation to specific MHC immune genes configuration in wildlife populations.

(c) *Allele frequency estimation*. MHC characterization and allele frequency estimation over larger scales (e.g., > 1000 km; latitude/altitude, see Cortázar‐Chinarro et al. (2017), as an example) might explain spatial patterns of genetic diversity over space and time. While the estimation of long‐term changes in allele frequency parameters remains quite challenging, model‐based approaches (e.g., Wang (2022)) could facilitate the estimation of allele frequencies. For example, developing model‐based approaches could help predict allele frequency changes under different evolutionary pressures, parasite pressures, and/or under different climatic scenarios.

There are limitations in current methodologies to characterize the MHC (Cortazar‐Chinarro et al. 2023; Gaigher et al. 2016; Lighten et al. 2014), as well as statistical limitations (Gaigher et al. 2019), as an example. One limitation of current amplicon‐based approaches is the difficulty of understanding the underlying MHC genomic architecture. MHC class I and class II gene copies and organization vary widely across taxa. Multiple gene copies are generated by gene duplication and diversity is amplified by gene conversion (Gaigher et al. 2016). Primers designed to capture diversity at the peptide binding domains of MHC molecules often amplify multiple loci and may show amplification bias between loci. Sequenced MHC allelic variants cannot be reliably assigned to MHC loci (Cortazar‐Chinarro et al. 2023).

Recent advancements in long‐read sequencing technologies (e.g., PacBio) have improved accuracy and reduced costs, providing researchers with valuable tools to better characterize the genetic architecture of the MHC in non‐model species (Dilthey 2021; Mellinger et al. 2023). These innovative technologies can be utilized by sequencing a subset of samples with long reads to lay the groundwork for MHC characterization, followed by population‐wide, amplicon‐based studies of MHC diversity or by MHC typing via long reads (Cheng et al. 2022). Capturing variation across the entire MHC gene complex could be achieved using a pangenomes approach, which involves high‐quality genomes to enable comparisons of these target immune genetic regions across organisms from different geographic areas (Chin et al. 2023). However, this approach currently entails significantly higher costs, and the availability of analytical pipelines for its implementation remains limited.

**FIGURE 2:**
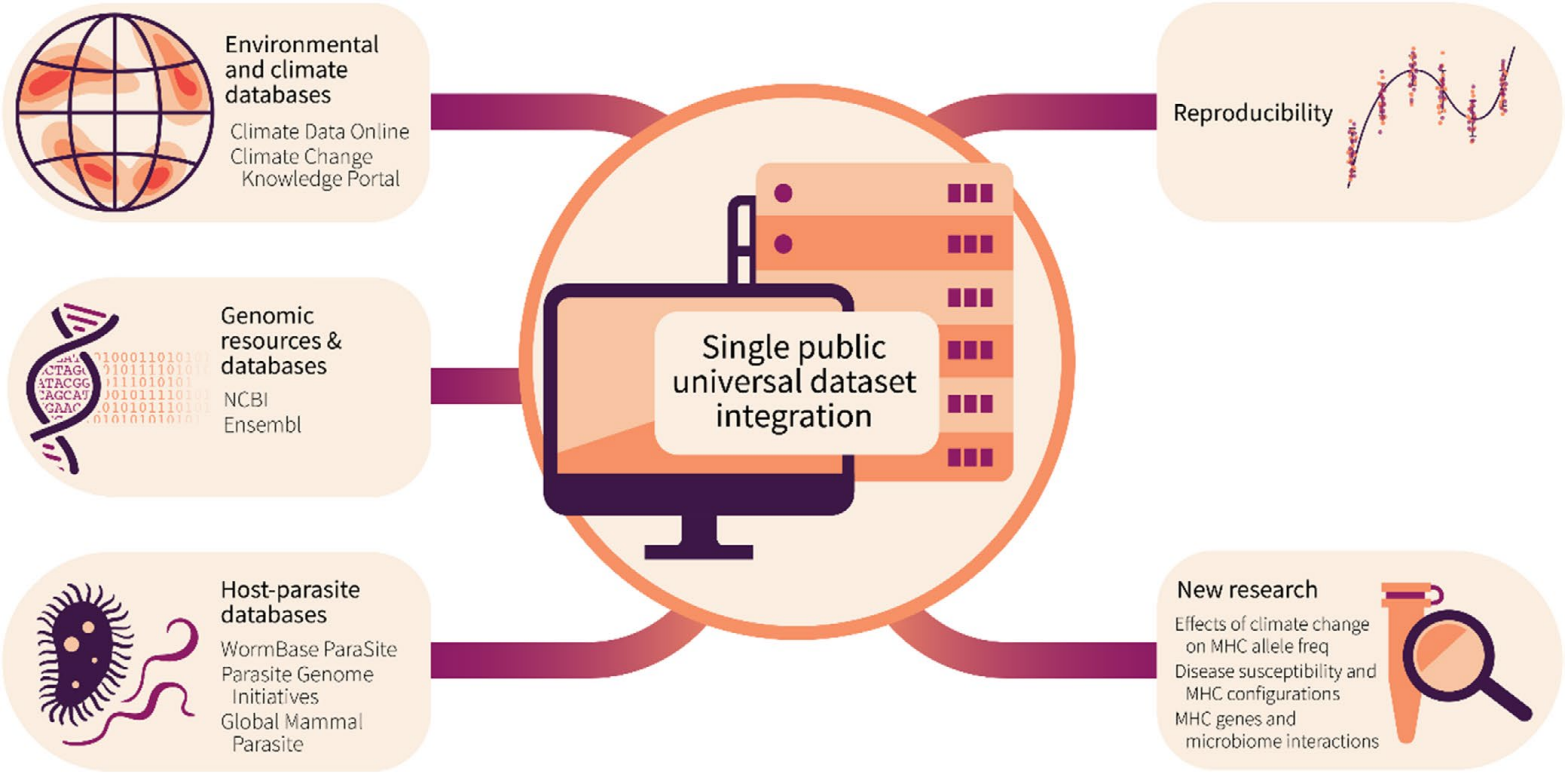
Multidisciplinary data integration diagram. Single public universal dataset integration would include multi‐disciplinary (e.g., environmental, genomic resources, and host–parasite interactions) databases contributing to reproducibility and the recycling of data for new projects in the field of immunogenetics.

Significant advancements have been made in eco‐immunology, enhancing our understanding of disease resistance and its relationship with immune defenses in wild animals. Most of these studies, however, focus on specific time points rather than offering temporally continuous and geographically diverse insights into host–parasite interactions. The lack of genomic data from many different species in relation to disease susceptibility or resistance is a challenge for addressing questions related to MHC characterization studies. This gap might be addressed by investing in public databases, giving the possibility of directly using genomic‐wide data generated previously into new research projects on genetic variation and infection. Such an approach would allow for working with large‐scale data and ensuring reproducibility. Standardized large data sets could be centralized and re‐used in future projects tackling the relationship between MHC diversity and parasitism (see Figure 2). Currently, large genomic data sets are already available from different international platforms such as NCBI (National Center for Biotechnology Information) or Ensembl genome browser. The potential integration of genomic data with other meta‐data (e.g., SILVA rRNA, parasitic organisms panels or climatic datasets) can be very complicated, despite the role for other factors in conferring resistance to infection (Ford and King 2016; Wolinska and King 2009). There is often a lack of consensus data in relation to genome version availability or file formats with the additional problem of expired links provided in publications. Using a single large, centralized dataset would make it easier to target complete genetic regions from well‐annotated genomes and transcriptomes. For example, it could easily target the MHC complex family genes or even a group of different immune regions within genomes of non‐model organisms. According to (Kosch et al. 2022), focusing on specific genetic regions alone may not provide a comprehensive vision of all immunogenetic effects and individual survival when carrying an infectious disease. Data science focused on extracting and applying the knowledge from typically large data sets would be optimal for immunogenetics, parasitic organisms and/or climate big data set integration to resolve these host–parasite evolutionary questions, particularly for wild systems. Data set integration would particularly help to answer the following questions:

1. How can climate shape MHC allele frequencies across geographic and temporal scales?
2. To what extent are changes in the allele frequencies of immune genes associated with host–parasite interactions and stochastic climatic events?
3. What are the follow‐on fitness implications for patterns of infectious disease in the wild?

## 5 Conclusions

Investigating mechanisms that maintain genetic diversity will advance our understanding of local adaptive processes, particularly from infectious diseases. Ongoing research of parasite‐driven selective processes under natural conditions should focus on characterizing MHC genetic variation at complete class I and II loci from well‐assembled and well‐annotated genomes and transcriptomes. In addition, changes in adaptive versus neutral marker allele frequencies over time should contribute to understanding the relative contribution of drift, selection, and parasite‐mediated selection mechanisms in shaping patterns of functional diversity. To estimate allele frequencies by using candidate and neutral markers, we must consider broad spatial and temporal scales across vertebrates as a whole. Furthermore, changes in allele frequency could be significant in habitats susceptible to sudden climatic events or the arrival of new parasites. These advances will only be possible by improving the accessibility of centralized public and multidisciplinary large data sets to increase our integrative knowledge on the importance of adaptive genetic variability in wild animals. Wildlife conservation efforts that monitor MHC genetic diversity across space and time—at the edges of species distributions, in long‐term fragmented habitats, or at northern latitudes—will be most effective for ensuring long‐term species preservation in the face of climate change and emerging diseases.

## Author Contributions

**Maria Cortazar‐Chinarro:** conceptualization (lead), funding acquisition (lead), investigation (lead), resources (lead), writing – original draft (lead). **Kayla C. King:** conceptualization (supporting), validation (supporting), visualization (supporting), writing – review and editing (supporting). **Mette Lillie:** conceptualization (equal), supervision (equal), validation (equal), writing – review and editing (supporting).

## Conflicts of Interest

The authors declare no conflicts of interest.

## Data Availability

The authors have nothing to report.
